# Problem or solution: The strange story of glucagon

**DOI:** 10.1016/j.peptides.2017.11.013

**Published:** 2018-02

**Authors:** R.V. Scott, S.R Bloom

**Affiliations:** Imperial College London, 6th Floor, Commonwealth Building, Hammersmith Hospital, London, W12 0NN, United Kingdom

**Keywords:** Glucagon, Diabetes, Obesity, Energy expenditure, Incretin, Oxyntomodulin

## Abstract

Globally, 13% of the world’s adult population is obese, and more than 400 million people suffer from diabetes. These conditions are both associated with significant morbidity, mortality and financial cost. Therefore, finding new pharmacological treatments is an imperative. Relative hyperglucagonaemia is seen in all types of diabetes, and has been implicated in its pathogenesis. Consequently, clinical trials are underway using drugs which block glucagon activity to treat type 2 diabetes. Conversely, exogenous glucagon can increase energy expenditure. Therefore, researchers are designing peptides that combine activation of the glucagon receptor with further incretin properties, which will treat obesity while mitigating the hyperglycaemic effects of glucagon. This review will discuss these conflicting physiological properties of glucagon, and the attempts to harness these effects pharmacologically.

## Highlights

• Hyperglucagonaemia is seen in all forms of diabetes including type 2 diabetes and alloxan-induced pancreatic destruction.
• Agents that block activity at the glucagon receptor are being used in clinical trials to treat diabetes.
• Peptides are being developed which combine activation of glucagon and incretin receptors to treat obesity.

## 1 Introduction

Glucagon as a subject of study had an inauspicious start. It was discovered as an impurity in early preparations of insulin, a ‘toxic fraction’ causing a rise in blood glucose and even death [1], [2]. Kimball and Murlin named it glucagon (GLUCose-AGONist) after a series of experiments designed to concentrate and isolate pure insulin found a precipitant which increased blood glucose in depancreatized dogs. However it took a further 25 years before Sutherland and De Duve purified glucagon itself [3].

Glucagon is a 29 amino acid peptide produced by the alpha cells in the pancreas. It is produced by proconvertase 2 processing products of the pre-pro-glucagon gene. Classically hypoglycaemia triggers glucagon release. Hypoglycaemia is sensed within the hypothalamus, particularly the ventromedial hypothalamic nucleus [4], [5], and the parasympathetic nervous system relays the signal to the pancreas to cause glucagon release [6], [7], [8]. The sympathoadrenal response to hypoglycaemia also stimulates glucagon release [7], [9], and intra-islet glucose levels affect glucagon production as well [9]. Glucagon release is inhibited by hyperglycaemia, insulin, GLP-1 and somatostatin [10], [11], [12]. Glucagon acts via a specific G-protein coupled receptor, which has wide-spread expression throughout the body, being particularly abundant in the liver, kidney, heart and adipose [13].

**Fig. 1:**
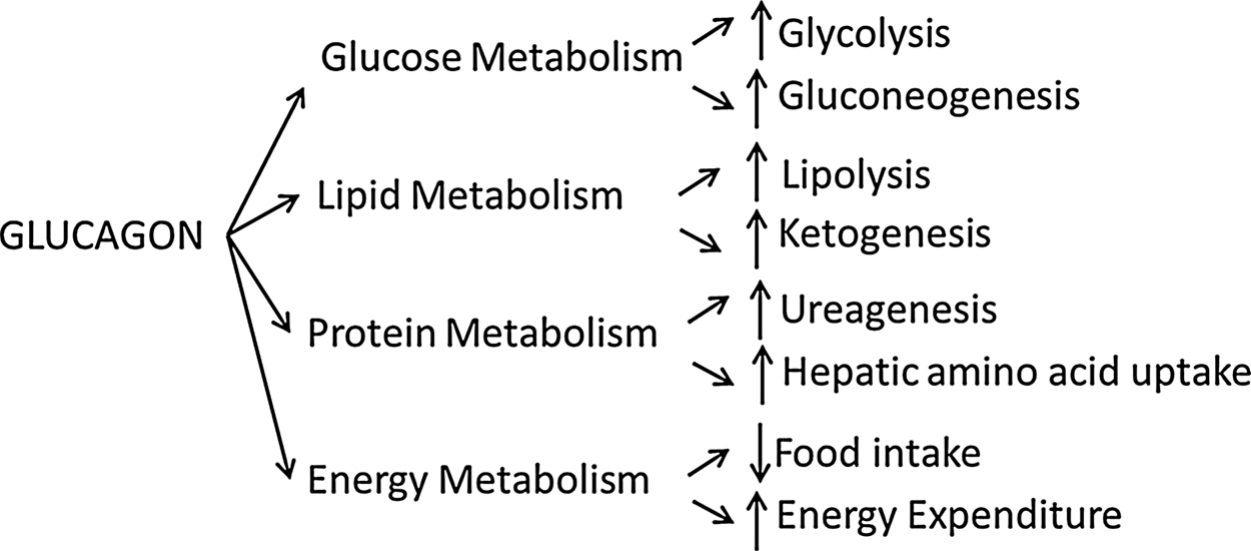
Summary of the metabolic effects of glucagon.

The main function of glucagon is to increase blood glucose, through both glycogenolysis and increased gluconeogenesis. It also affects lipid metabolism, breaking down fat through lipolysis and increasing ketone production [14]. Glucagon affects protein metabolism, increasing ureagenesis and causing amino acid uptake into hepatocytes [15], [16], [17]. The resultant carbon skeletons can then enter the gluconeogenic pathway. Glucagon therefore acts in multiple ways to maintain fuel supply to all organs in the body (Fig. 1).

## 2 The bihormonal hypothesis

It was recognised in the 1920s that insulin deficiency was the cause of diabetes, and that administration of pancreatic extracts containing insulin could successfully treat the hyperglycaemia [18], [19]. In contrast, glucagon appeared to have little significant function in disease, and its clinical use was limited to rare occasions of reversing the effects of insulin [20], [21]. Then in 1973, Roger Unger and Lelio Orci proposed the bihormonal-abnormality hypothesis of diabetes, stating that glucagon elevation was as important as insulin deficiency [22]. This rooted glucagon as a central problem in the disease.

Glucagon has been found to be elevated in all forms of diabetes, from alloxan-induced diabetes in dogs, to patient with type 1 and type 2 diabetes; even following pancreatectomy [23], [24], [25], [26], [27], [28], [29]. Physiological studies have also demonstrated that glucagon has opposing effects to insulin on carbohydrate, fat and protein metabolism, with insulin causing glycogenesis, lipid formation and having an anabolic effect on muscle [30], [31], [32], [33], [34], [35], [36], [37]. The bihormonal-abnormality hypothesis combines these two findings, stating that the relative glucagon excess and insulin deficiency, plus the opposing actions of these two hormones, leads to the hyperglycaemia of diabetes.

The bihormonal hypothesis was supported by studies that showed that suppression of glucagon with somatostatin limited the hyperglycaemia seen in patients with type 1 diabetes and alloxan-diabetic dogs [38], [39], [40]. Over the following 40 years, further evidence was produce by studies using glucagon-receptor knock-out mice. Compared to wild-type mice, *Gcgr-/-* mice have constitutively lower blood glucose levels, improved glucose homeostasis and a lean phenotype [41]; they are also resistant to the hyperglycaemic and hyperinsulinaemic effects of high-fat diet, and following beta-cell destruction with streptozotocin, have normal glucose levels and improved response to glucose challenges, as well as lower levels of gluconeogenic enzymes, all markers of improved diabetic control [42], [43]. Moreover, if the glucagon receptor is restored with adenovirus, the diabetic profile is returned [44]. However, recent studies have shown that while preventing glucagon activity can mitigate the effects of insulin deficiency, this only works if there is some residual insulin signalling left. In absolute insulin deficiency, due to either complete beta-cell destruction or insulin gene knockout, glucagon action blockade does not prevent hyperglycaemia [45], [46], [47].

### 2.1 Glucagon receptor antagonists

Nevertheless, the bihormonal hypothesis has made blockade of the glucagon receptor a potential treatment for diabetes. Preclinical studies have supported numerous different approaches Table 1. Glucagon receptor antagonists improve glucose-mediated blood glucose excursions in mice including diabetic models [48], [49], [50]. Glucagon receptor antibodies reduce baseline glucose levels and improve glucose tolerance in diabetic rodents and monkeys [51], [52], [53], [54]; they also reduce the hepatic expression of gluconeogenic enzyme mRNA [51]. Anti-sense oligonucleotides which reduce expression of the hepatic glucagon receptor also improve glucose levels and improve glucose tolerance in diabetic mice [55]. Even glucagon-neutralizing L-RNA aptamers (Spiegelmers) have been developed, which improve glucose excursions following IPGTTs in diabetic mouse models [56].

**Table 1:** Classes of drugs being developed which target glucagon activity to treat obesity and diabetes.

Agents developed to block activity at the glucagon receptor to treat diabetes	Glucagon-receptor co-agonists developed to treat obesity
Glucagon receptor antagonists [48], [49], [50], [57], [58], [59], [60], [61]	Glucagon/GLP-1 co-agonists [85], [86], [87], [88], [89], [90]
Antibodies against the glucagon receptor [51], [52], [53], [54], [65]	Glucagon/GLP-1/GIP tri-agonists [110], [111], [112]
Antisense oligonucleotides [55], [62], [63], [64]	T3 coupled to glucagon [113]
Glucagon-neutralizing Spiegelmers [56]	

The success of these pre-clinical studies has inevitably led to trials in humans of agents which reduce activity at the glucagon receptor. These trials have confirmed that this is an effective approach for treatment of diabetes. Glucagon receptor antagonists improve fasting and post-prandial blood glucose levels, as well as HbA1c [57], [58], [59], [60], [61]. Antisense oligonucleotides also improve HbA1C in people with diabetes in phase 2 clinical trials [62], [63], [64] while monoclonal antibodies against the glucagon receptor reduce glucagon-induced glucose excursions [65].

Each of these classes of glucagon blocking drugs has been associated with significant side effects. Increased hepatic transaminases have been seen with the antisense oligonucleotides [64], glucagon receptor antagonists [57], [58], [59], [60], [66], [67] and humanized monoclonal antibodies [65]. The small molecule glucagon receptor antagonists also increase LDL cholesterol, a highly undesirable side effect given the association of increased cholesterol, type 2 diabetes and cardiovascular disease [60], [61], [67], [68]; and one, LY2409012, causes an increase in hepatic fat fraction [66]. All can exaggerate a fall in blood sugar, with the potential for serious hypoglycaemia, though the number of symptomatic hypoglycaemic episodes actually seen in clinical trials is low [57], [58], [66], [67]. Pre-clinical studies have also shown that glucagon receptor antibodies cause compensatory alpha cell hyperplasia [51], [52]. What clinical effect this has long-term has not yet been ascertained, but there is a concern that this hyperplasia may become malignant. These side effects have stymied the development of several GRAs (including MK-3577 and MK-0893), and indeed there are no anti-diabetic agents in current clinical practice which work by blocking glucagon activity. Nevertheless, several agents are still in the developmental pipeline, and the results of further clinical trials are awaited.

### 2.2 Glucagon and obesity

Much research has focused on blocking the action of glucagon to treat diabetes since the articulation of the bi-hormonal abnormality hypothesis. However, there has been recent interest in using glucagon to treat obesity, and subsequently treat type 2 diabetes through weight loss.

The hyperglycaemic effects of glucagon were first noted in the 1920s. It wasn’t until the 1950s that glucagon was found to have other metabolic effects. In 1957, Schulman et al. showed that glucagon reduced appetite, and could even cause weight loss in man [69]. Then in 1960, Salter showed through a pair-feeding paradigm that glucagon causes an increase in energy expenditure in rodents [70], an increase that was confirmed by indirect calorimetry [71]. Subsequently, several studies in man have shown that an infusion of glucagon can increase energy expenditure as well as reduce food intake [71], [72], [73], [74]. This combination of effects makes glucagon a very attractive anti-obesity treatment, as typically drugs which increase energy expenditure also cause an increase in food intake [75], [76], which means there is likely to be no overall loss of weight; and conversely, but equally problematically for an obesity treatment, reducing food intake is usually accompanied by a decrease in energy expenditure, with subsequent limits as to the weight which can be lost [77].

### 2.3 Glucagon/incretin receptor dual agonists

The question remained, however, as to how to harness these effects of glucagon without causing harmful hyperglycaemia. The answer arrived following studies using the hormone oxyntomodulin.

Oxyntomodulin is a 37 amino-acid peptide, consisting of a glucagon sequence with an octapeptide tail. It is an alternative product of the pre-pro-glucagon gene, produced by proconvertase 1 in the L-cells of the ileum. There is no known specific oxyntomodulin receptor, but it does activate both the glucagon receptor and GLP-1 receptors. Oxyntomodulin has successfully been used in clinical trials to cause a significant loss of weight in both normal and overweight subjects [78], [79]. It does this both through reducing food intake [78], [79], [80], [81], [82] and through increasing energy expenditure [79], [81]. Moreover, this weight loss is not accompanied by any deterioration in glucose tolerance [78], [79].

GLP-1 analogues are already in commercial use as treatments for diabetes, and relatively recently, the analogue liraglutide has been licensed as a stand-alone therapy for obesity (Saxenda, Novo Nordisk). However, after an initial dramatic reduction in body weight, the weight loss tails off [83]. This may be because of a reduction in metabolic rate which accompanies the weight loss [84]. Furthermore, GLP-1 and its analogues cause significant nausea, and many patients stop the drugs due to gastrointestinal side effects, a phenomenon which limits the doses that can be tolerated. Therefore, co-administration of glucagon with GLP-1 would allow both hormones to be administered at relatively low doses, enabling the beneficial weight loss effects of both hormones to be felt, without the adverse side effects.

This theory has been confirmed in numerous studies. Tan et al. found a 45 min infusion of glucagon with GLP-1 caused a significant increase in energy expenditure which was identical to the increase seen with glucagon alone, but the GLP-1 blunted the rise in glucose seen with glucagon on its own [73]. Cegla et al. showed that co-infusion of glucagon with GLP-1 at doses which, alone, were sub-anorectic, combined to cause a significant reduction in food intake and an absolute increase in energy expenditure, but without the significant rise in blood glucose which was seen with the glucagon infusion alone [74].

As such, there is interest in developing glucagon-GLP-1 co-agonists (i.e. an oxyntomodulin analogue) to treat obesity. Numerous rodent studies have shown that long-acting oxyntomodulin analogues can cause weight loss [85], [86], [87], [88], [89]. These work through both a reduction in food intake [85], [86], [87], [89] and increased energy expenditure. This is associated with a loss of fat mass. Moreover, acutely these drugs reduce glucose excursion following a glucose challenge [86], [87], [90]. Chronic administration has been shown to improve glucose homeostasis in diabetic mouse models [85], [87], [88], [89].

The success of these pre-clinical studies has meant that at least 11 different oxyntomodulin analogues are currently in development as potential treatments for obesity [91]. However, critical questions still need to be answered about these drugs. Though the glucagon component of the analogues is responsible for the increased energy expenditure [92], the physiological processes which lead to this increased energy expenditure are still undetermined. Though early studies suggested glucagon increased brown adipose tissue (BAT) activity [93], [94], [95], [96], these studies all used indirect measures of BAT activity. No studies have shown that peripherally administered glucagon increases UCP-1 levels, the molecular marker of BAT activity. Furthermore there is direct evidence that BAT is not activated by glucagon, with no increase in BAT temperature following a glucagon infusion despite an increase in energy expenditure [72]. Other drugs which increase energy expenditure, such as thyroxine, dinitrophenol and amphetamines, have significant side effects, it is therefore desirable that the underlying mechanism behind the oxyntomodulin effect is understood.

The effects of chronic glucagon agonism are unclear. Glucagon affects protein metabolism, enhancing hepatic uptake of amino acids [17], [97], [98], [99], upregulating urea production [100], [101], [102], and causing hypoaminoacidaemia [15], [103]. Glucagonoma patients, with chronic high levels of glucagon, have increased whole-body protein breakdown [104] with a clinically typical skin rash cured with amino acid supplementation. There are situations where endogenous oxyntomodulin is chronically elevated, notably after gastric bypass surgery and in short bowel syndrome [105], [106], [107], [108]; there is no evidence that the elevated hormone levels is detrimental in either of these conditions, and indeed may be responsible for improved weight loss and diabetic control seen after bariatric surgery [106], [107]. Nevertheless, enhanced catabolism is unlikely to be helpful so the impact of oxyntomodulin analogues on protein metabolism needs to be investigated.

The success of glucagon/GLP-1 co-agonists has led to studies looking at whether other hormones can be combined with glucagon to treat obesity Table 1. Glucose-dependent insulinotropic peptide (GIP) has incretin properties [109]. Tri-agonist analogues, which activate the glucagon, GLP-1 and GIP receptors, have been developed, with the aim that the additional GIP activity will further offer better protection from glucagon-induced hyperglycaemia. Pre-clinical studies have confirmed that these analogues can reduce body weight in obese mice models [110], [111], [112] as well as improve blood glucose with chronic administration, even if acutely the glucagon component may cause a transient hyperglycaemia [111], [112]. Such multifunctional agents have the distinct disadvantage of having a fixed ratio of effect at each receptor, usually identified as appropriate in rodents, whereas human therapy may require a different ratio for optimal therapy. Comparison trials of such agents with oxyntomodulin analogues in man have not to date been published.

Glucagon has also been coupled to tri-iodothyronine [113]. Thyroid hormone has many effects on metabolism, including improving lipid profiles and increasing energy expenditure [114], [115], which has led to it being recommended previously as a treatment for obesity [116]. However, exogenous administration of thyroid hormones can cause significant side-effects, such as arrhythmias and osteopenia. By attaching tri-iodothyronine to a glucagon moiety, the thyroid hormone should be directed to areas where the glucagon receptor is expressed in high numbers, such as the liver and adipose tissue. Targeting the activity of thyroid hormone in this way should therefore improve hepatic lipid metabolism and cause a reduction in adiposity, without causing the cardiac and other side-effects.

Administration of a uni-molecular tri-iodothyronine/glucagon agonist did have beneficial metabolic effects, due to activity at both the glucagon and thyroid receptors. The dual agonist reduced plasma cholesterol to an equivalent degree as equimolar glucagon, while it reduced plasma triglycerides to a similar extent as T3; hepatic cholesterol levels were reduced more by the dual agonist than either single hormone. Overall there was also a greater reduction in body weight with the dual agonist than either single hormone; the glucagon component prevented the hyperphagia that accompanied the T3 administration, while T3 caused an increase in EE. Tri-iodothyronine can also potentiate insulin signalling [117]. The addition of thyroid activity to glucagon improved glucose tolerance acutely, as well as after 7 days of administration, negating the hyperglycaemic effects of glucagon alone. Directing the thyroid hormone to the liver did improve side effects: the tachycardia and cardiac hypertrophy seen with T3 were avoided with the dual-agonist, as was the reduction in bone volume seen with T3, validating the technique to minimize the side-effects of systemic tri-iodothyronine use.

## 3 Conclusions

The studies discussed here show two distinct roles for glucagon in the treatment of diabetes. Glucagon may have a direct role in the pathogenesis of diabetes, with a relative hyperglucagonaemia in all forms of diabetes. It is not clear if this is a consequence or a cause of diabetes, nor whether harmful or a possible endogenous counter-regulation mechanism to compensate for some adverse metabolic change. Antagonising glucagon action may reduce the associated hyperglycaemia, but may not be beneficial in terms of life expectation of the person with diabetes. One of the main causes of type 2 diabetes is obesity. Glucagon can cause significant weight loss through reducing food intake and increasing energy expenditure; and if this reduces obesity, could itself treat diabetes. To mitigate any hyperglycaemic effects of the glucagon, it will, however, have to be administered with another hormone such as GLP-1 which releases insulin and thereby inhibits the enhanced gluconeogenesis of glucagon.

Despite promising experimental results, neither of these approaches, either glucagon agonism or antagonism, is at present used in clinical practice. Glucagon antagonists, either as small molecules or antibodies against the glucagon receptor, are in clinical trials, but need to have improved safety profiles to progress to license. Oxyntomodulin analogues are also being developed, but their therapeutic advantage might depend on optimizing the relative activity at the glucagon and GLP-1 receptors, balancing the increased energy expenditure and reduced food intake with effects on blood glucose. Moreover, the long-term effects of these drugs remain to be investigated. Determining whether glucagon is understood to be a problem in diabetes which needs to be suppressed, or a solution to the global obesity epidemic, is likely to depend on which approach is successfully brought to market first, rather than the relative merits of the two approaches. It might be both!

## Funding

The Section of Endocrinology and Investigative Medicine is funded by grants from the MRC, BBSRC, NIHR, an Integrative Mammalian Biology (IMB) Capacity Building Award, an FP7- HEALTH- 2009- 241592 EuroCHIP grant and is supported by the NIHR Biomedical Research Centre Funding Scheme. The views expressed are those of the author(s) and not necessarily those of the funders, the NHS, the NIHR or the Department of Health. R. Scott is also funded by the Wellcome Trust.
